# Persistence of CMV-specific anti-HIV CAR T cells after adoptive immunotherapy

**DOI:** 10.1128/jvi.01933-24

**Published:** 2025-04-10

**Authors:** Chengxiang Wu, Nathan M. Johnson, Shan Yu, Agnes S. Lo, Gautam K. Sahu, Preston A. Marx, Dorothee von Laer, Gail Skowron, Romas Geleziunas, George M. Shaw, Amitinder Kaur, Richard P. Junghans, Stephen E. Braun

**Affiliations:** 1 Tulane National Primate Research Center, Tulane University School of Medicine 12255, Covington, Louisiana, USA; 2 Department of Immunology, Tulane University School of Medicine 12255, New Orleans, Louisiana, USA; 3 Tufts Medical School, Boston, Massachusetts, USA; 4 Roger Williams Medical Center 21186, Providence, Rhode Island, USA; 5 Department of Tropical Medicine, School of Public Health and Tropical Medicine, Tulane University 25812 , New Orleans, Louisiana, USA; 6 Medizinische Universität Innsbruck 27280, Innsbruck, Austria; 7 Gilead Sciences 57037, Foster City, California, USA; 8 Division of Hematology/Oncology, University of Pennsylvania 312087 , Philadelphia, Pennsylvania, USA; 9 IT Bio, LLC., Boston, Massachusetts, USA; 10 Department of Pharmacology, Tulane University School of Medicine 12255, New Orleans, Louisiana, USA; University of Virginia, Charlottesville, Virginia, USA

**Keywords:** adoptive immunotherapy, CD4-CAR T cells, CMV-specific immune responses, persistence and biodistribution, rhesus SHIV-challenge model

## Abstract

The success of chimeric antigen receptor (CAR)-T cell (Tc) immunotherapy in refractory B-cell acute lymphoblastic leukemia (B-ALL) suggests adaptation of this strategy toward HIV. Because cytomegalovirus (CMV) vaccine vectors generated Tc responses that controlled viral replication, these studies aim to genetically modify CMV-specific Tc with HIV-CAR2 vectors and link HIV immunotherapy to persistent CMV antigen stimulation. To mimic a clinical scenario, rhesus macaques were challenged with the CCR5-tropic simian/human immunodeficiency virus (SHIV-D) prior to antiretroviral therapy (ART). Autologous CMV-specific Tc were transduced with the control CEA-CAR2 or CD4-CAR2/maC46 vectors and reinfused. After stopping ART, the plasma viral load (PVL) in the control rebounded and was sustained above 1.7 × 10^4^ copies/mL; PVL in CD4-CAR2-treated animals was delayed up to 6 weeks and 10-fold lower. The CD4 CAR-Tc frequency peaked at day 7 and was detected in lymphoid tissues at 6 weeks. Both CEA-CAR2 and CD4-CAR2 persisted in PBMCs for about 2 years, which indicates that the CMV-specific CAR Tc were maintained based on their CMV specificity. However, long-term PVL was stable in all animals. Thus, CMV-specific CAR-Tc were active initially, persisted long term, but failed to control viral replication.

**IMPORTANCE:**

Because of latent viral reservoirs and a dysfunctional immune response, HIV replication rebounds when antiretroviral therapy is interrupted. Therefore, cytomegalovirus (CMV)-specific Tc were genetically modified with anti-HIV CD4-CAR2 vectors to link the targeting of the HIV envelope to the persistent CMV immune response. In this clinical scenario with simian/human immunodeficiency virus (SHIV) challenge and antiretroviral therapy (ART) suppression, early activity of the CAR Tc delayed rebound in the rhesus macaque/SHIV challenge model. However, even with long-term persistence of CAR Tc in the blood, control of viral replication was not achieved. These data suggest that CAR Tc will require additional interventions to cure HIV infection.

## INTRODUCTION

In untreated HIV-infected individuals, both HIV-specific and total CD8^+^ T lymphocytes are dysfunctional (1–7). While combination antiretroviral therapy (ART) controls HIV replication and improves this immune dysfunction (8–10), persistent viral reservoirs still lead to viral rebound when the treatment is interrupted (11). Thus, HIV-specific CD8^+^ T lymphocytes induced by natural infection are unable to suppress viral replication after discontinuing ART and may require additional interventions to improve their functional activity. Although the initial trials with CD4-CAR T cells in HIV-infected individuals using only TCRζ intracellular signaling failed to control viral replication (12–14), the next generation of HIV therapeutic strategies is being developed, including adoptive T cell immunotherapy (15).

Since a decrease in HIV-1 plasma viral load (PVL) with ART is associated with a decrease in the magnitude and breadth of HIV-specific CD8^+^ T lymphocyte responses (1–7), the level of antigen expression during ART may be insufficient to maintain bulk CAR Tc in an activated state with cytolytic function. In contrast, cytomegalovirus (CMV)-specific T lymphocytes in asymptomatic CMV-seropositive individuals are known to persist at high frequencies during the lifetime of their host; moreover, they typically maintain an effector memory phenotype, are distributed among peripheral tissues, and lack features of T cell exhaustion (16, 17). The frequency of CMV-specific CD8^+^ T lymphocytes can continue to expand even in the absence of detectable CMV viremia (16, 17). Additionally, recombinant replication-competent rhesus CMV vaccine vectors expressing SIV antigens induce SIV-specific cellular immune responses capable of controlling SIV replication in rhesus macaques (18, 19). Therefore, these current studies were designed to link the HIV-specific CAR2 vector to the CMV-specific T cell response as a means of providing long-term immunosurveillance of simian/human immunodeficiency virus (SHIV) in tissue reservoirs.

## RESULTS

**Fig 1:**
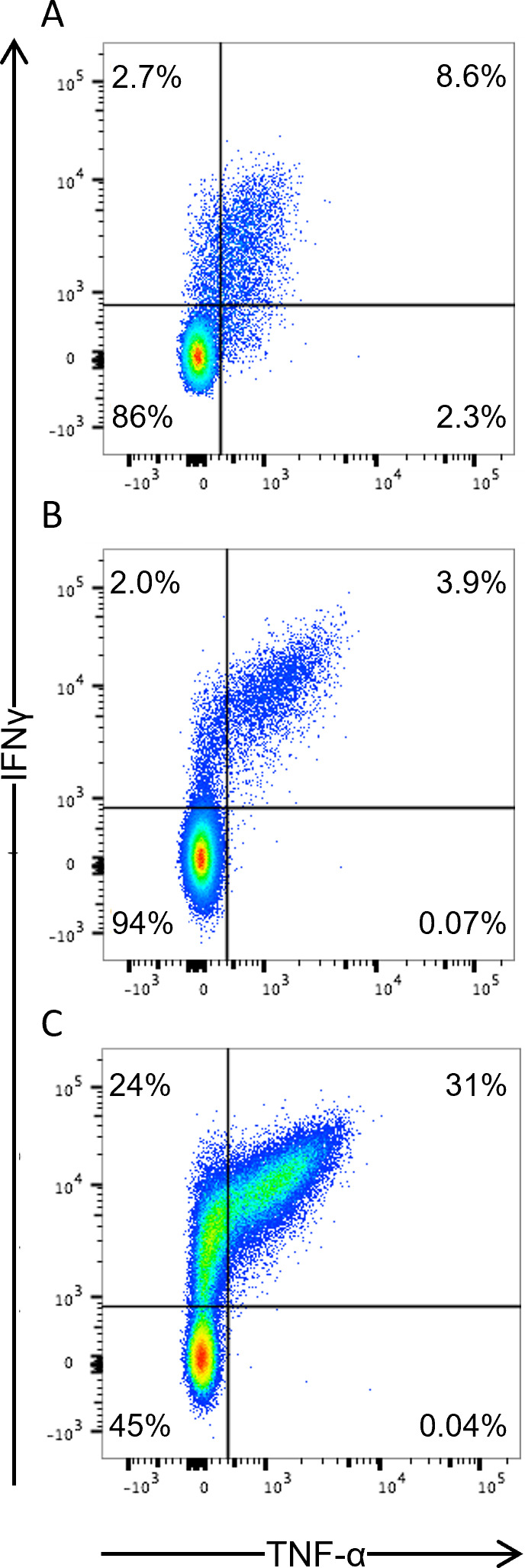
The frequency of rhesus CMV-specific CD8^+^ T lymphocytes in (A) PBMCs *ex vivo,* (B) bead-stimulated, and (C) CMV peptide-stimulated populations by intracellular cytokine staining. PBMCs were stimulated with CD3/CD28-coated beads (B) or rhesus CMV peptide pools (C) and then expanded for 2 weeks with IL-2. Unstimulated (A) or expanded cells were restimulated with CMV peptides plus anti-CD49d and anti-CD28 costimulatory antibodies overnight in the presence of brefeldin A and then stained for intracellular IFN-γ/TNF-α production. Cells were gated for singlets, live cells, lymphocytes, CD3^+^ cells, and CD8^+^ cells.

To demonstrate the feasibility of expanding CMV-specific T lymphocyte populations in rhesus macaque Old World monkeys (see Fig. S1 at https://doi.org/10.25833/r1m2-qn67), we stimulated rhesus PBMCs with three rhesus CMV peptide pools (IE1, IE2, and pp65) along with anti-CD28 antibody and 100 U per mL of IL-2. As controls, PBMCs were also stimulated with anti-CD3/anti-CD28 antibodies cross-linked to beads and 100 IU per mL of IL-2. After 12 days of culture, the frequency of CMV-specific CD8^+^ T lymphocytes in the CMV peptide-expanded cells was compared with the CD3/CD28-expander bead-cultured cells as well as with *ex vivo* uncultured PBMCs. In cryopreserved PBMCs from this rhesus macaque, 13.6% of CD8^+^ T lymphocytes produced IFNγ and/or TNFα cytokines *ex vivo* following overnight stimulation with rhesus CMV peptide pools, with most cytokine-secreting CD8^+^ T lymphocytes producing both cytokines (Fig. 1A). After bulk T cell expansion with CD3/CD28 beads, the frequency of CMV-specific CD8^+^ T lymphocytes in the cultured cells was not increased from that present in *ex vivo* uncultured PBMCs (Fig. 1B). In contrast, rhesus CMV peptide-stimulated cells showed an increase in the frequency of single or dual IFN-γ/TNF-α, CMV-specific, CD8^+^ T lymphocytes from 13.6% to 55% (Fig. 1C), with the majority of activated cells producing both cytokines. The total number of CMV-specific CD4^+^ lymphocytes also increased by 100-fold (data not shown), but the majority of CMV-responsive cells in culture were CD8^+^ T lymphocytes. These data demonstrate the utility of the CMV IE1, IE2, and pp65 peptides for the specific expansion of multifunctional rhesus CMV-specific T lymphocytes.

**Fig 2:**
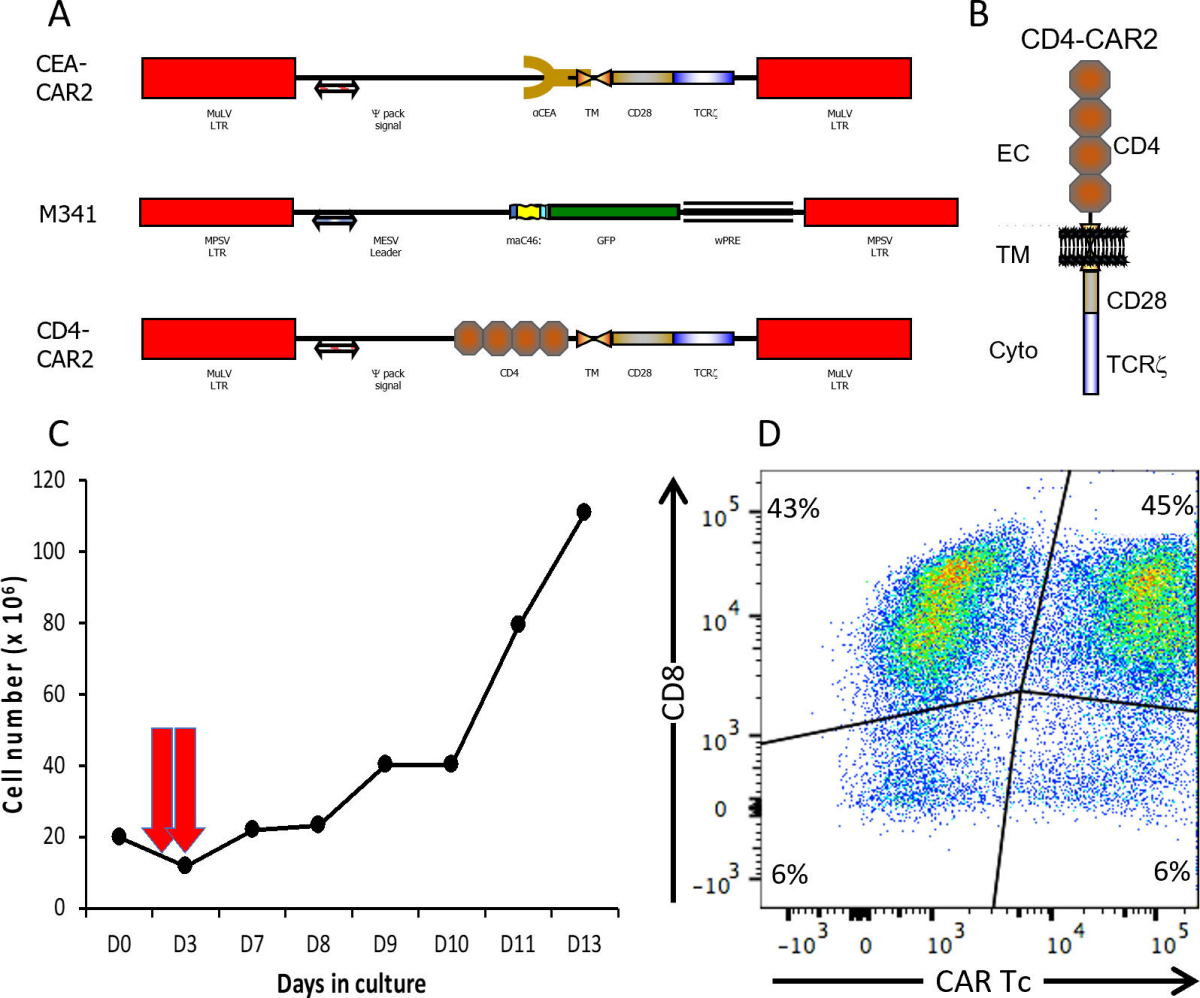
Transduction of CMV-specific Tc with retroviral vectors. (A) Schematic diagrams of the vector expressing control CAR (αCEA-CAR2), maC46 (M341), and the αHIV-CAR (CD4-CAR2). (B) Structure of the CD4-CAR2 with the D1-D4 CD4 extracellular (EC) domains, the CD28 transmembrane (TM) domain, and CD28/TCRζ cytoplasmic signaling domains. (C) Clinical-scale expansion and transduction of rhesus CMV-specific T cell populations. Autologous rhesus PBMCs were stimulated with CMV overlapping peptide pools (IE1, IE2, and pp65) and CD28 antibody plus IL-2 (50 IU/mL) and IL-15 (50 ng/mL). On Days 2 and 3, cells were exposed to the retroviral vector coated on retronectin (20 µg/cm^2^) and expanded for 10 more days. (D) Expression of the CAR vector in CD8^+^ T lymphocytes by flow cytometry.

To genetically modify the autologous CMV-specific T cells with CAR2 vectors (Fig. 2A) for adoptive transfer into the rhesus SHIV challenge model, we stimulated rhesus PBMCs in serum-free media (see Fig. S2 at https://doi.org/10.25833/r1m2-qn67) with the three rhesus CMV peptide pools (IE1, IE2, and pp65) plus CD28 antibody (see Fig. S3 at https://doi.org/10.25833/r1m2-qn67) and IL-2/IL-15 (see Fig. S4 at https://doi.org/10.25833/r1m2-qn67) and exposed cells to the gammaretroviral vectors on days 2 and 3 (Fig. 2C). We used the CD4-CAR2 vector to redirect rhesus CMV-specific T lymphocytes toward HIV-infected cells; these cells were co-transduced with the membrane-associated C46 (maC46) fusion inhibitor (20, 21) to protect cells from SHIV infection. The CD4-CAR2 utilizes the four extracellular immunoglobulin domains of CD4, the CD28 transmembrane domain, and the CD28 and TCRζ intracellular signaling domains (Fig. 2B) (22). As a control, we transduced rhesus CMV-specific T lymphocytes with the anti-carcinoembryonic antigen (CEA)-CAR2 vector (Fig. 2A) (23). Large-scale culture of the cells expanded up to 120 × 10^6^ cells (Fig. 2C). Most of the cells are CD8^+^ (88%), and more than 50% of the cells express the CAR on the cell surface (Fig. 2D).

**Fig 3:**
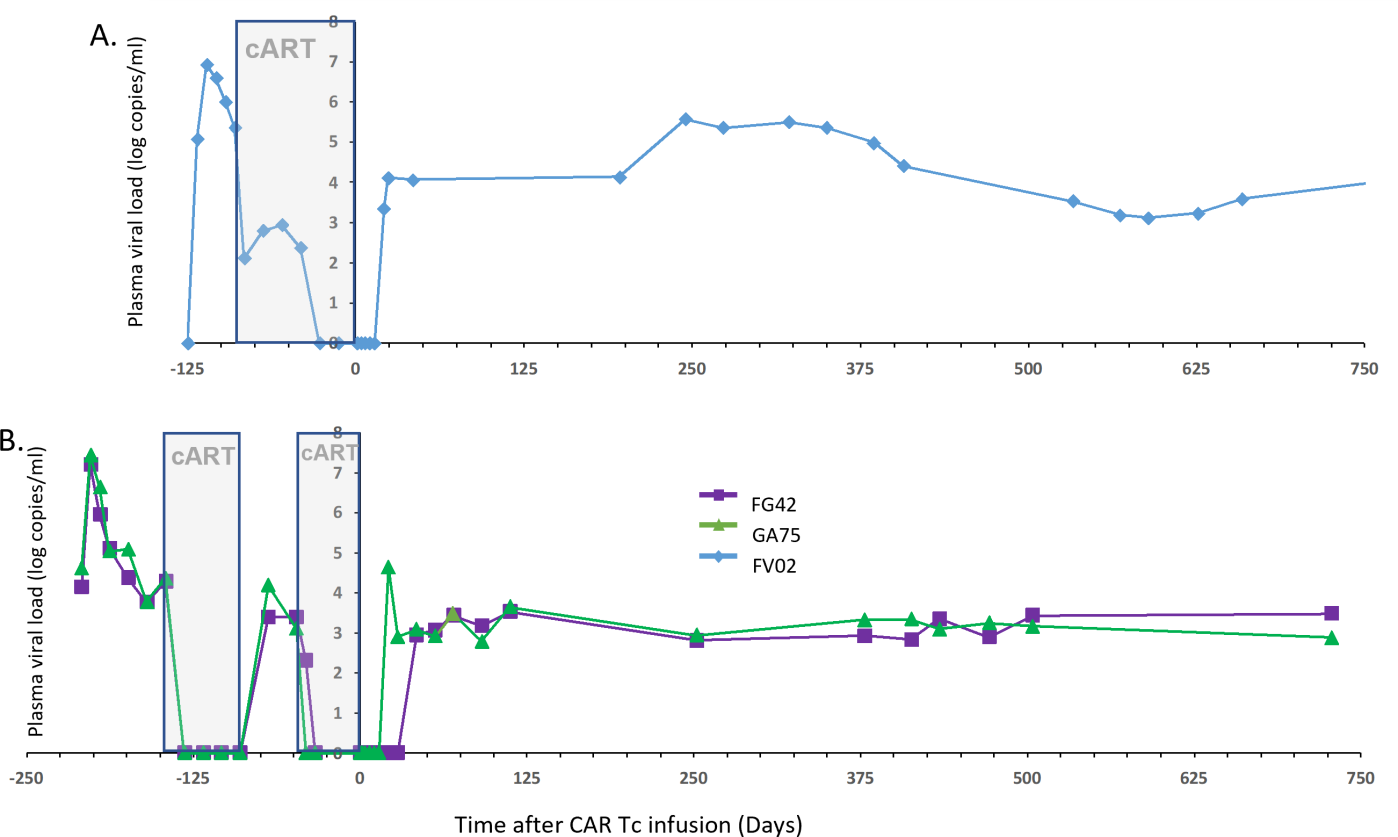
Plasma viral load before and after CAR2 Tc immunotherapy. To mimic a clinical scenario, rhesus macaques were challenged with the CCR5-tropic chimeric SHIV-D (50 ng SIV Gag p27 per animal) and administered ART (tenofovir 20 mg/kg/day; emtricitabine 20 mg/kg/day; dolutegravir 2.5 mg/kg/day) subcutaneously (gray shaded). At time 0, (A) animal FV02 (blue diamond) received the αCEA-CAR2 control-transduced CMV-specific T lymphocytes; (B) animals FG42 (purple square) and GA75 (green triangle) received CD4-CAR2/maC46 transduced CMV-specific T lymphocytes. Plasma was collected longitudinally and assessed for viral replication by qRT-PCR.

To simulate a clinical scenario for adoptive immunotherapy, we challenged rhesus macaques with the CCR5-tropic chimeric HIV/SIV (SHIV-D) (24) and followed the plasma viral load (PVL) (Fig. 3). Similar to HIV-1 in humans, SHIV PVL peaked at approximately 10^7^ RNA copies per mL on day 14 and lowered to the viral set point by week 6 post-infection. Animals were administered triple-drug combination anti-retroviral therapy (ART, gray boxes), which included daily injections of the nucleotide reverse transcriptase inhibitors tenofovir (20 mg/kg) and emtricitabine (30 mg/kg) and the integrase inhibitor dolutegravir (2.5 mg/kg), to reduce the PVL to undetectable levels (Fig. 3). As an internal control for viral rebound, ART was interrupted for 6 weeks in two animals, and PVL rebounded to the set point levels near 3,500 copies per mL by 3 weeks (Fig. 3B). ART was administered to reduce PVL to undetectable levels prior to adoptive Tc therapy.

**Fig 4:**
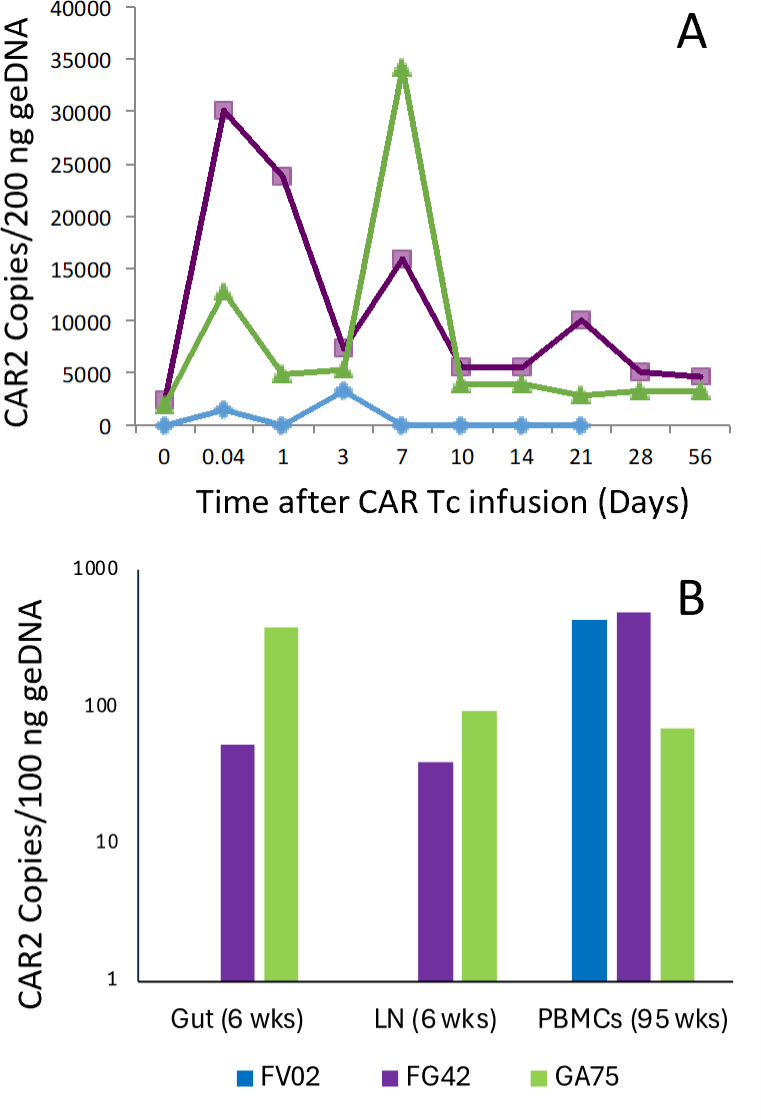
Vector copy number in PBMCs and lymphoid tissues. Genomic DNA from lymphoid tissues was isolated from (A) acute PBMCs at various time points and from (B) both gut and LN at 6 weeks and PBMCs at 95 weeks after CAR2 Tc infusion in FG42, GA75 (CD4-CAR2 Tc), FV02 (CEA-CAR2), or negative control (grey) animals and then assessed for the vector-copy number by qPCR.

CMV-specific T cells transduced with CAR2 vectors were transferred to the SHIV-challenged/ART-treated autologous host; then ART was stopped, and lymphoid tissue samples were collected longitudinally to assess CAR2 Tc biodistribution and PVL. The FV02 control received 110 × 106 ^C.E.^ CEA-CAR2 T cells; while FG42 and GA75 received 46.7 and 34.8 × 10^6^ CD4-CAR2 T cells, respectively. Since the animal experiments were overlapping but not concurrent, the FV02 control samplings extend beyond the FG42 and GA75 samplings. As shown in Fig. 4, the CD4-CAR2 Tc spiked in the peripheral blood of FG42 and GA75 after adoptive transfer, as detected by qPCR, where the level of the CD4-CAR2 was significantly higher (1.5–3.0 × 10^4^ copies) on day 7 than the peak copies of the CEA-CAR2 (2,500 copies) in FV02 on day 3 (Fig. 4A). Additionally, the quantity of the therapeutic CD4-CAR2 vector in the PBMC increased from day 3 to day 7 (Fig. 4A), which may have resulted from the proliferation of CD4-CAR2 Tc *in vivo*. In lymph nodes and gut biopsies collected 6 weeks after adoptive transfer, the CD4-CAR2 was detectable in both FG42 and GA75 at around 0.5% and 2% of total cells (Fig. 4B), which may indicate homing and immunosurveillance of SHIV reservoirs by the CMV-specific CD4-CAR2 Tc.

The PVL was also assessed during the immediate interval after adoptive transfer (Fig. 3). In comparison to the rebound in PVL during the structured treatment interruption (STI) prior to CAR2 Tc immunotherapy, the rebound in PVL was similar in GA74 (at 3 weeks) and delayed in FG42 to 6 weeks (set point of 1.4 × 10^3^ copies per mL) after adoptive transfer of the CMV-specific CD4-CAR2 Tc. Additionally, the increase in PVL in GA75 peaked and then receded to the stable set point (1.4 × 10^3^ copies per mL), compatible with some degree of immunological control over viral replication. The rebound in PVL in FV02 control animals was also delayed by 3 weeks, and the set point was 1.7 × 10^4^ copies per mL; we do not have an STI prior to adoptive transfer to compare in this animal. Therefore, the delays in PVL could also be a normal variation in the kinetics of viral rebound.

To assess the long-term effects of CMV-specific CAR Tc, we collected samples for about 2 years. The frequency of therapeutic and control CAR Tc in the PBMCs was stabilized between 0.5% and 2% of all cells after more than 95 weeks (Fig. 4B). Similar levels of control and therapeutic vectors *in vivo* suggest that long-term maintenance is based predominantly on the persistent CMV antigen presentation rather than on the CAR T cell signaling. However, even with long-term stable levels of CD4-CAR2 Tc in PBMCs, the set point PVL was unaltered (Fig. 3).

## DISCUSSION

These studies demonstrate the feasibility of expanding and genetically modifying autologous CMV-specific T cells with CD4-CAR2 vectors for adoptive immunotherapy. We show that these cells can engraft in lymphoid tissues and persist *in vivo* for almost 2 years. Long-term persistence of the CAR Tc appears to be due to the CMV specificity and not by stimulation through the CAR2 signaling domains since the CEA-CAR2 control vector persisted at similar levels. Viral rebound was delayed in one animal treated with the therapeutic vectors compared with STI in the same animal prior to adoptive CAR T cell transfer, and the viral setpoints were slightly lower than those of the control animal for the duration of the study. Concurrently, the CD4-CAR2 Tc expanded slightly *in vivo* after adoptive transfer, although additional studies will be necessary to conclude that these changes result directly from the functional activity of CD4-CAR2. We found that CMV-specific T cells can be used to manufacture CAR2 Tc, which migrate to lymphoid reservoirs after adoptive transfer and persist for almost 2 years *in vivo*.

The CD4-CAR2 expresses the CD4 extracellular domain to redirect CTL activity against HIV Env and the CD28 and TCRζ intracellular T cell signaling domains to stimulate CTL functions. Using the CD4 domain to target HIV envelope binding would limit the selection of mutant SHIV strains as the loss of binding to CD4 would reduce viral fitness. These cells are co-transduced with the maC46 (20), a potent fusion inhibitor to protect modified cells from SHIV infection. Although the CD4-CAR2 target cells express Env (25), the long-term persistence of the modified cells is based on CMV antigen stimulation because even the CEA-CAR2 Tc maintained equivalent levels of vector copies in the PBMCs at 95 weeks. These CMV-specific Tc respond to rhCMV IE1, IE2, and pp65 (26), and expression of these antigens in latent cells may contribute to increase in the CMV-specific Tem cells (27, 28), and the persistent detection of CAR2 Tc after adoptive transfer (Fig. 4). Viral-specific T cells have been used for adoptive therapy to control other viral infections (CMV, EBV, AdV, and others) in the context of HSCT (29–31) or even genetically modified to enhance or redirect targeting (28, 32–35). In these viral infections, reactivation of viremia or increased viral antigen expanded the CAR Tc and increased their functional activity (28, 36). Although the CAR2 vector expresses CD28 and TCRζ, how the intracellular signaling contributes to Tc memory differentiation or the lack of exhaustion in CMV-specific Tc has not been determined. Other signaling domains such as CD27, OX40, 4–1BB, or IL2/15R (37–39) may better support expansion of the CAR Tc in the context of lentiviral infection; however, only a few studies have utilized NHP models (40–43) or clinical trials (44) to evaluate these CAR vectors. In our study, the CD4-CAR2 Tc increased homing or proliferation in the LN and gut at 6 weeks (Fig. 4), even though the antigen load in the tissues is expected to be low during ART. In contrast, the control CEA-CAR2 Tc was not detectable in these tissues at this time point, indicating that the CD4-CAR2 Tc may respond to viral antigens in the SHIV reservoir or as the virus reactivates. For an effective cure strategy, the HIV-specific CD8^+^ T cell responses need to be superior to those elicited by natural infection; however, viral replication appears to exceed the capacity of the CD4-CAR2 Tc to control viral rebound. Expression of HIV Env may be too low for the CD4-CAR2 Tc, and a latency-reactivating agent may be required while animals remain on ART (45).

This study explores the potential for CMV-specific Tc to maintain immunologic surveillance of viral reservoirs and provide implications for current clinical trials in patients with HIV (46) or for trials in patients with solid tumors. This effort mimics a clinical scenario (viral infection, ART suppression, and then CAR Tc therapy) in the rhesus challenge model—an important model from which to judge the safety and efficacy of immunotherapies. While active initially without evident toxicity, these CMV-specific CAR Tc failed to control viral replication and will require more potent interventions.

## MATERIALS AND METHODS

### CMV screen of animal subjects

A group of six rhesus macaques were screened for reactivity to three rhesus CMV peptide pools (15 aa overlapping 11); IE1 (115 peptides), IE2 (149 peptides), and pp65 (134 peptides), using ELISpot Pro Monkey IFN-γ assay (MANTECH 3420M-2APT-2). These three peptides were chosen as they are major CMV viral proteins targeted by adaptive cellular responses (47). For stimulation of the cells with CMV peptides, 300,000 cells per well were incubated for 24 hours with 0.8 μg/mL of the peptide pools. IFN-γ spots were detected according to the manufacturer’s protocol. Spots were counted in a blinded fashion by ZellNet Consulting (Fort Lee, NJ) using a KS ELISpot reader system (Zeiss, Thornwood, NY) with KS ELISpot software Version 4.9.16, following international guidelines for ELISpot plate evaluation (48). Three animals with strong responses to at least one of the peptides (see Fig. S1 at https://doi.org/10.25833/r1m2-qn67) were selected, and PBMCs from the three animals were collected and stored in liquid nitrogen to receive genetic modification.

### SHIV-D challenge/antiretroviral therapy

This study was conducted at the Tulane National Primate Research Center (TNPRC), which is fully AAALAC-accredited, using purpose breed Indian Rhesus macaque. All procedures were IACUC-approved and were performed in accordance with the Guide for the Care and Use of Laboratory Animals. The SHIV model, an SIV backbone with the HIV gp120 envelope, is necessary for the purposes of these studies as the maC46 fusion inhibitor specifically targets HIV-1 Env. We chose SHIV-D because the challenge of rhesus macaques had previously demonstrated that most animals maintain a stable viral set point (24). SHIV-D is R5 tropic and similar to human strains in infecting CD4^+^ CCR5^+^ T_EM_ cells and leading to CD4 depletion in the gut mucosa (49). It shows a clinically relevant viral setpoint and is resistant to spontaneous control. Recruited animals were challenged i.v. with 50 ng HIV Gag p24 from transient transfection of HEK293T cells with the SHIV-D construct (24).

After PVL reached a set point (chronic steady-state PVL), animals were administered the antiretroviral therapy regimen consisting of the nucleotide reverse transcriptase inhibitor tenofovir (TFV) 20 mg/kg, the nucleoside reverse transcriptase inhibitor emtricitabine (FTC) 30 mg/kg, and the integrase inhibitor dolutegravir (DTG) 2.5 mg/kg. Once fully suppressed, ART therapy was temporarily suspended to track the kinetics of viral rebound in plasma. When animals reached plateau levels of circulating viral RNA, ART was reinitiated to lower PVL to undetectable levels until adoptive transfer of autologous T cells.

### Plasma viral load detection

Plasma viral load was assayed by qPCR throughout the duration of the experiment by the TNPRC qPCR core lab with a sensitivity of 1.9 Equivalent Viral Copies log (Eq. VC log) or 83 copies/mL of plasma (50).

### Gammaretroviral vectors

Vectors are MLV-based gammaretroviral vectors (Fig. 2A). Anti-HIV CAR2 was constructed with the four human extracellular immunoglobulin domains (D1–D4) from CD4, the transmembrane and intracellular signaling domain of human CD28, and the intracellular signal transduction domain of human CD3ζ chain. (Fig. 2B). The M341 expresses the membrane-associated C46 fusion inhibitor (maC46) with a human IgG_2_ hinge and CD34 transmembrane domain fused to intracellular GFP (kind gift from DVL) (Fig. 2B). The constructed anti-HIV-1 CAR2 and the maC46::GFP were subcloned into the MFG vector backbone created by Richard C. Mulligan at the Harvard Gene Therapy Institute (51). The αCEA CAR2 control vector was previously described (52).

### Vector production

HEK293FT cells (Invitrogen) were cultured with Dulbecco’s modified Eagle’s medium (DMEM) (ThermoFisher) supplemented with 10% heat-inactivated fetal bovine serum (FBS; GIBCO, USA) and 1% penicillin/streptomycin (Gibco, USA). To prepare high-titer retroviral vectors, around 2 × 10^6^ 293 FT cells were seeded in T-75 flasks 24 hours before transfection through an optimized calcium phosphate-mediated transfection protocol (53) with minor modifications. Briefly, 2–4 hours before the transfection, the culture medium was replaced with 14 mL fresh medium in each T-75 flask. To prepare the transfection cocktail, 28 µg of the retroviral transfer plasmid containing CAR2, 4 µg of the pCMV-VSV-G plasmid, and 8 µg pCMV-MLV-gag-pol were mixed to a final volume of 500 μL with 0.25 M calcium chloride. An equal volume of 2 x HBS is added to the mix dropwise while vortexing. The transfection cocktail is incubated at room temperature for 3–5 minutes and distributed evenly to the media of a T-75 flask. Retroviral supernatants containing either the therapeutic or anti-CEA control vectors were PEG-concentrated (54) overnight at 4°C, centrifugated at 1500 x *g* for 30 minutes, and resuspended in 500 µL RPMI1640 medium without serum.

### Isolation, stimulation, transduction, and expansion of CMV-specific T cells

To isolate peripheral blood mononuclear cells (PBMCs), peripheral blood from rhesus macaques was collected in EDTA tubes, spun, and separated using a discontinuous density-gradient centrifugation (LSM) (Lonza, Lake Charles, LA), processed with ACK lysis buffer to remove residual RBCs and cryopreserved. To stimulate PBMCs, cells were stimulated with anti-CD3/CD28 beads (Stem Cell Technology, Vancouver, Canada) at a cell:bead ratio of 1:2 in 10 mLs or three rhesus CMV peptide pools (rhCMV IE1 pool with 115 peptides, rhCMV IE2 pool with 149 peptides, and the rhCMV pp65 with 134 peptides) at 0.8μg/mL plus anti-CD28 antibody (1 μg/mL). For the large-scale stimulation, PBMCs (2.0 × 10^7^) were resuspending in 650 µL R10 (RPMI1640 supplemented with 10% heat-inactivated FBS, GlutaMax, and 50 IU/mL penicillin and 50 µg/mL streptomycin), mixed with 650 µL R10 with each peptide pool (5 ng per peptide) and CD28 antibody, incubated at 37°C for 1 hour, and mixed every 15 minutes. Following the incubation, cells and peptide mix are further diluted with 10 mL CTS OpTmizer T Cell Expansion SFM (ThermoFisher Scientific) supplemented with penicillin/streptomycin and 50 IU/mL IL-2 (R&D systems) and 50 ng/mL IL-15 (R&D systems).

To transduce stimulated PBMCs, 24-well non-tissue culture-treated plates were coated with 20 µg per well retronectin (Takara Bio, Fisher Scientific) overnight at 4°C. Plates were washed three times with PBS on the day of transduction, blocked with R10 for 30 minutes at room temperature, and preloaded with viral particles by centrifuging the supernatant at 500 x *g* for 60 minutes at 30°C. Following 3 days stimulation, non-viable cells were removed through a separation with LSM and plated at 1 × 10^6^ cells/mL on the retronectin-coated, viral-preloaded plates with an additional viral supernatant. The plates were spun at 500 x *g* for 60 minutes at 30°C to enhance transduction efficiency and then cultured in media plus cytokines for 10–14 days at 37°C, 5% CO_2_. The total cell number was determined. Cells were washed with PBS, resuspended in 10 mLs PBS, and injected into autologous animals intravenously over 10 mins.

### Intracellular cytokine responses and flow cytometry

The anti-CD3/CD28 bead- or CMV peptide-expanded lymphocytes were cultured without IL-2 overnight and then added into FACS tubes coated with 10 µg/mL of the anti-CD49d mAb monoclonal antibodies in the presence or absence of a final concentration of 0.5 µg/mL anti-CD3 (clone 6G12, NHP resource) or CMV-specific stimulating antigens consisting of rhesus CMV peptide pools of overlapping 15 amino acid (aa) peptides spanning the rhesus CMV immediate early 1 (IE1), IE2, pp65 (26). The prestimulated cells were also stimulated with 20 ng/mL PMA and 1 µg/mL ionomycin as the positive controls. Cryopreserved PBMCs without prestimulation were treated with the same conditions and used as controls. Cells were incubated at 37°C in a 5% CO_2_ incubator for 1 hour, and then 1 µL/mL brefeldin A (Golgi Plug) was added to each tube and incubated for another 12 hours.

Cells (0.5 x 10^6^–2 × 10^6^ cells per sample) were stained with the following antibodies (see Table S1 at https://doi.org/10.25833/r1m2-qn67) and for surface and intracellular cytokine markers using standard protocols (26). Briefly, the cultured cells were washed with PBS supplemented with 2% fetal calf serum and stained with the LIVE/DEAD Fixable Aqua Dead Cell Stain Kit (Invitrogen, Grand Island, NY) and fluorochrome-conjugated monoclonal antibodies speciﬁc for cell surface markers (Table S1). Cells were then washed and ﬁxed by incubation with FIX & PERM Medium A (Caltag Labs, Burlingame, CA) for 15 minutes at room temperature, followed by permeabilization with FIX & PERM Medium B (Caltag Labs, Burlingame, CA) along with intracellular antibodies for IFNγ, TNFα, and CD69. Samples were acquired on an LSR II or Fortessa flow cytometer (BD Biosciences, San Jose, CA), and the data were analyzed using FlowJo software (Tree Star, Ashland, OR). IFNγ and TNFα expression levels were evaluated by measuring the frequency of cytokines in each activated T lymphocyte subset (expressing CD69).

### Quantitative PCR

Primers for amplification of CAR2 vectors such as the forward (5′-GCAAGCATTACCAGCCCTAT-3′) and reverse (5′-GTTCTGGCCCTGCTGGTA-3′) primers were designed with melting temperature within 2°C; and the probe (5′-ATCGCTCCAGAGTGAAGTTCAGCA-3′) contained the FAM fluorescent reporter and Black Hole Quencher (BHQ) for added specificity. Cycle conditions were initial denaturation at 95°C for 10 minutes, followed by 40 cycles at 95°C for 15 seconds and 64°C for 1 minute. All reactions use Taqman Universal Mastermix (Fisher 4364338) and run on ThermoFisher 7900HT. The albumin gene (81 bps) was amplified with the forward (5- accatgcttttcagctctgg-3) and reverse (5- tctgcatggaaggtgaatgt-3) primers to quantitate genomic DNA. Amplifications were started at 50°C for 2 minutes and 95°C for 20 seconds, 40 cycles at 95°C for 3 seconds and 60°C for 30 seconds, followed by the melting curve stage of one cycle of 95°C for 15 seconds, 60°C for 1 minutes, 95°C for 15 seconds, and 60°C for 15 seconds. Real-time PCR was performed using the 7500 Fast Real-Time PCR system (Applied Biosystems). Product specificity was confirmed by melt curve analysis and gel electrophoresis. Absolute CAR copies and WBC numbers were calculated from plasmid standard curves.

## Data Availability

Supplemental material is available at https://doi.org/10.25833/r1m2-qn67. Materials and data that are reasonably requested by others will be made available in a timely fashion, at reasonable cost, and in limited quantities to members of the scientific community for noncommercial purposes, in compliance with all Material Transfer Agreements.

## References

[1] Betts MR, Ambrozak DR, Douek DC, Bonhoeffer S, Brenchley JM, Casazza JP, Koup RA, Picker LJ. 2001. Analysis of total human immunodeficiency virus (HIV)-specific CD4^+^ and CD8^+^ T-cell responses: relationship to viral load in untreated HIV infection. J Virol 75:11983–11991. doi:10.1128/JVI.75.24.11983-11991.2001 11711588 PMC116093

[2] Eller MA, Goonetilleke N, Tassaneetrithep B, Eller LA, Costanzo MC, Johnson S, Betts MR, Krebs SJ, Slike BM, Nitayaphan S, Rono K, Tovanabutra S, Maganga L, Kibuuka H, Jagodzinski L, Peel S, Rolland M, Marovich MA, Kim JH, Michael NL, Robb ML, Streeck H. 2016. Expansion of inefficient HIV-specific CD8 T cells during acute infection. J Virol 90:4005–4016. doi:10.1128/JVI.02785-15 26842474 PMC4810544

[3] Lichterfeld M, Kaufmann DE, Yu XG, Mui SK, Addo MM, Johnston MN, Cohen D, Robbins GK, Pae E, Alter G, Wurcel A, Stone D, Rosenberg ES, Walker BD, Altfeld M. 2004. Loss of HIV-1-specific CD8^+^ T cell proliferation after acute HIV-1 infection and restoration by vaccine-induced HIV-1-specific CD4^+^ T cells. J Exp Med 200:701–712. doi:10.1084/jem.20041270 15381726 PMC2211961

[4] Nasi A, Chiodi F. 2018. Mechanisms regulating expansion of CD8+ T cells during HIV-1 infection. J Intern Med 283:257–267. doi:10.1111/joim.12722 29315893

[5] Ndhlovu ZM, Kamya P, Mewalal N, Kløverpris HN, Nkosi T, Pretorius K, Laher F, Ogunshola F, Chopera D, Shekhar K, Ghebremichael M, Ismail N, Moodley A, Malik A, Leslie A, Goulder PJR, Buus S, Chakraborty A, Dong K, Ndung’u T, Walker BD. 2015. Magnitude and kinetics of CD8^+^ T cell activation during hyperacute HIV infection impact viral set point. Immunity 43:591–604. doi:10.1016/j.immuni.2015.08.012 26362266 PMC4575777

[6] Perdomo-Celis F, Taborda NA, Rugeles MT. 2019. CD8^+^ T-cell response to HIV infection in the era of antiretroviral therapy. Front Immunol 10:1896. doi:10.3389/fimmu.2019.01896 31447862 PMC6697065

[7] Warren JA, Clutton G, Goonetilleke N. 2019. Harnessing CD8^+^ T cells under HIV antiretroviral therapy. Front Immunol 10:291. doi:10.3389/fimmu.2019.00291 30863403 PMC6400228

[8] Bushman F, Landau NR, Emini EA. 1998. New developments in the biology and treatment of HIV. Proc Natl Acad Sci USA 95:11041–11042. doi:10.1073/pnas.95.19.11041 9736685 PMC33895

[9] Carr A. 2003. Toxicity of antiretroviral therapy and implications for drug development. Nat Rev Drug Discov 2:624–634. doi:10.1038/nrd1151 12904812

[10] Nabatanzi R, Cose S, Joloba M, Jones SR, Nakanjako D. 2018. Effects of HIV infection and ART on phenotype and function of circulating monocytes, natural killer, and innate lymphoid cells. AIDS Res Ther 15:7. doi:10.1186/s12981-018-0194-y 29544508 PMC5853105

[11] Hoen B, Fournier I, Lacabaratz C, Burgard M, Charreau I, Chaix M-L, Molina J-M, Livrozet J-M, Venet A, Raffi F, Aboulker J-P, Rouzioux C, PRIMSTOP Study Group. 2005. Structured treatment interruptions in primary HIV-1 infection: the ANRS 100 PRIMSTOP trial. J Acquir Immune Defic Syndr 40:307–316. doi:10.1097/01.qai.0000182628.66713.31 16249705

[12] Mitsuyasu RT, Anton PA, Deeks SG, Scadden DT, Connick E, Downs MT, Bakker A, Roberts MR, June CH, Jalali S, Lin AA, Pennathur-Das R, Hege KM. 2000. Prolonged survival and tissue trafficking following adoptive transfer of CD4ζ gene-modified autologous CD4^+^ and CD8^+^ T cells in human immunodeficiency virus–infected subjects. Blood 96:785–793. doi:10.1182/blood.V96.3.785 10910888

[13] Walker RE, Bechtel CM, Natarajan V, Baseler M, Hege KM, Metcalf JA, Stevens R, Hazen A, Blaese RM, Chen CC, Leitman SF, Palensky J, Wittes J, Davey RT Jr, Falloon J, Polis MA, Kovacs JA, Broad DF, Levine BL, Roberts MR, Masur H, Lane HC. 2000. Long-term in vivo survival of receptor-modified syngeneic T cells in patients with human immunodeficiency virus infection. Blood 96:467–474. doi:10.1182/blood.V96.2.467 10887107

[14] Deeks SG, Wagner B, Anton PA, Mitsuyasu RT, Scadden DT, Huang C, Macken C, Richman DD, Christopherson C, June CH, Lazar R, Broad DF, Jalali S, Hege KM. 2002. A phase II randomized study of HIV-specific T-cell gene therapy in subjects with undetectable plasma viremia on combination antiretroviral therapy. Mol Ther 5:788–797. doi:10.1006/mthe.2002.0611 12027564

[15] Su H, Mueller A, Goldstein H. 2024. Recent advances on anti-HIV chimeric antigen receptor-T-cell treatment to provide sustained HIV remission. Curr Opin HIV AIDS 19:169–178. doi:10.1097/COH.0000000000000858 38695148 PMC11981014

[16] Klenerman P, Oxenius A. 2016. T cell responses to cytomegalovirus. Nat Rev Immunol 16:367–377. doi:10.1038/nri.2016.38 27108521

[17] Karrer U, Sierro S, Wagner M, Oxenius A, Hengel H, Koszinowski UH, Phillips RE, Klenerman P. 2003. Memory inflation: continuous accumulation of antiviral CD8^+^ T cells over time. J Immunol 170:2022–2029. doi:10.4049/jimmunol.170.4.2022 12574372

[18] Hansen SG, Vieville C, Whizin N, Coyne-Johnson L, Siess DC, Drummond DD, Legasse AW, Axthelm MK, Oswald K, Trubey CM, Piatak M Jr, Lifson JD, Nelson JA, Jarvis MA, Picker LJ. 2009. Effector memory T cell responses are associated with protection of rhesus monkeys from mucosal simian immunodeficiency virus challenge. Nat Med 15:293–299. doi:10.1038/nm.1935 19219024 PMC2720091

[19] Wilson NA, Reed J, Napoe GS, Piaskowski S, Szymanski A, Furlott J, Gonzalez EJ, Yant LJ, Maness NJ, May GE, et al.. 2006. Vaccine-induced cellular immune responses reduce plasma viral concentrations after repeated low-dose challenge with pathogenic simian immunodeficiency virus SIVmac239. J Virol 80:5875–5885. doi:10.1128/JVI.00171-06 16731926 PMC1472612

[20] Kimpel J, Braun SE, Qiu G, Wong FE, Conolle M, Schmitz JE, Brendel C, Humeau LM, Dropulic B, Rossi JJ, Berger A, von Laer D, Johnson RP. 2010. Survival of the fittest: positive selection of CD4^+^ T cells expressing a membrane-bound fusion inhibitor following HIV-1 infection. PLoS One 5:e12357. doi:10.1371/journal.pone.0012357 20808813 PMC2925957

[21] MacLean AG, Walker E, Sahu GK, Skowron G, Marx P, von Laer D, Junghans RP, Braun SE. 2014. A novel real-time CTL assay to measure designer T-cell function against HIV Env^+^ cells. J Med Primatol 43:341–348. doi:10.1111/jmp.12137 25138734 PMC4318253

[22] Sahu GK, Sango K, Selliah N, Ma Q, Skowron G, Junghans RP. 2013. Anti-HIV designer T cells progressively eradicate a latently infected cell line by sequentially inducing HIV reactivation then killing the newly gp120-positive cells. Virology (Auckl) 446:268–275. doi:10.1016/j.virol.2013.08.002 PMC3791854 24074590

[23] Ma Q, DeMarte L, Wang Y, Stanners CP, Junghans RP. 2004. Carcinoembryonic antigen-immunoglobulin Fc fusion protein (CEA-Fc) for identification and activation of anti-CEA immunoglobulin-T-cell receptor-modified T cells, representative of a new class of Ig fusion proteins. Cancer Gene Ther 11:297–306. doi:10.1038/sj.cgt.7700685 15002034

[24] Li H, Wang S, Kong R, Ding W, Lee F-H, Parker Z, Kim E, Learn GH, Hahn P, Policicchio B, et al.. 2016. Envelope residue 375 substitutions in simian-human immunodeficiency viruses enhance CD4 binding and replication in rhesus macaques. Proc Natl Acad Sci USA 113:E3413–E3422. doi:10.1073/pnas.1606636113 27247400 PMC4914158

[25] Yang OO, Tran AC, Kalams SA, Johnson RP, Roberts MR, Walker BD. 1997. Lysis of HIV-1-infected cells and inhibition of viral replication by universal receptor T cells. Proc Natl Acad Sci USA 94:11478–11483. doi:10.1073/pnas.94.21.11478 9326635 PMC23511

[26] Chan KS, Kaur A. 2007. Flow cytometric detection of degranulation reveals phenotypic heterogeneity of degranulating CMV-specific CD8^+^ T lymphocytes in rhesus macaques. J Immunol Methods 325:20–34. doi:10.1016/j.jim.2007.05.011 17628586 PMC2039909

[27] Trgovcich J, Kincaid M, Thomas A, Griessl M, Zimmerman P, Dwivedi V, Bergdall V, Klenerman P, Cook CH. 2016. Cytomegalovirus reinfections stimulate CD8 T-memory inflation. PLoS One 11:e0167097. doi:10.1371/journal.pone.0167097 27870919 PMC5117776

[28] Wang X, Wong CW, Urak R, Mardiros A, Budde LE, Chang W-C, Thomas SH, Brown CE, La Rosa C, Diamond DJ, Jensen MC, Nakamura R, Zaia JA, Forman SJ. 2015. CMVpp65 vaccine enhances the antitumor efficacy of adoptively transferred CD19-redirected CMV-specific T cells. Clin Cancer Res 21:2993–3002. doi:10.1158/1078-0432.CCR-14-2920 25838392 PMC4489991

[29] Gerdemann U, Katari UL, Papadopoulou A, Keirnan JM, Craddock JA, Liu H, Martinez CA, Kennedy-Nasser A, Leung KS, Gottschalk SM, Krance RA, Brenner MK, Rooney CM, Heslop HE, Leen AM. 2013. Safety and clinical efficacy of rapidly-generated trivirus-directed T cells as treatment for adenovirus, EBV, and CMV infections after allogeneic hematopoietic stem cell transplant. Mol Ther 21:2113–2121. doi:10.1038/mt.2013.151 23783429 PMC3831033

[30] Papadopoulou A, Gerdemann U, Katari UL, Tzannou I, Liu H, Martinez C, Leung K, Carrum G, Gee AP, Vera JF, Krance RA, Brenner MK, Rooney CM, Heslop HE, Leen AM. 2014. Activity of broad-spectrum T cells as treatment for AdV, EBV, CMV, BKV, and HHV6 infections after HSCT. Sci Transl Med 6:242ra83. doi:10.1126/scitranslmed.3008825 PMC4181611 24964991

[31] Bollard CM, Heslop HE. 2016. T cells for viral infections after allogeneic hematopoietic stem cell transplant. Blood 127:3331–3340. doi:10.1182/blood-2016-01-628982 27207801 PMC4929925

[32] Rossig C, Bollard CM, Nuchtern JG, Rooney CM, Brenner MK. 2002. Epstein-Barr virus-specific human T lymphocytes expressing antitumor chimeric T-cell receptors: potential for improved immunotherapy. Blood 99:2009–2016. doi:10.1182/blood.v99.6.2009 11877273

[33] Pule MA, Savoldo B, Myers GD, Rossig C, Russell HV, Dotti G, Huls MH, Liu E, Gee AP, Mei Z, Yvon E, Weiss HL, Liu H, Rooney CM, Heslop HE, Brenner MK. 2008. Virus-specific T cells engineered to coexpress tumor-specific receptors: persistence and antitumor activity in individuals with neuroblastoma. Nat Med 14:1264–1270. doi:10.1038/nm.1882 18978797 PMC2749734

[34] Louis CU, Savoldo B, Dotti G, Pule M, Yvon E, Myers GD, Rossig C, Russell HV, Diouf O, Liu E, Liu H, Wu M-F, Gee AP, Mei Z, Rooney CM, Heslop HE, Brenner MK. 2011. Antitumor activity and long-term fate of chimeric antigen receptor-positive T cells in patients with neuroblastoma. Blood 118:6050–6056. doi:10.1182/blood-2011-05-354449 21984804 PMC3234664

[35] Cruz CRY, Micklethwaite KP, Savoldo B, Ramos CA, Lam S, Ku S, Diouf O, Liu E, Barrett AJ, Ito S, Shpall EJ, Krance RA, Kamble RT, Carrum G, Hosing CM, Gee AP, Mei Z, Grilley BJ, Heslop HE, Rooney CM, Brenner MK, Bollard CM, Dotti G. 2013. Infusion of donor-derived CD19-redirected virus-specific T cells for B-cell malignancies relapsed after allogeneic stem cell transplant: a phase 1 study. Blood 122:2965–2973. doi:10.1182/blood-2013-06-506741 24030379 PMC3811171

[36] Lapteva N, Gilbert M, Diaconu I, Rollins LA, Al-Sabbagh M, Naik S, Krance RA, Tripic T, Hiregange M, Raghavan D, et al.. 2019. T-cell receptor stimulation enhances the expansion and function of CD19 chimeric antigen receptor-expressing T cells. Clin Cancer Res 25:7340–7350. doi:10.1158/1078-0432.CCR-18-3199 31558475 PMC7062259

[37] Lim RM, Rong L, Zhen A, Xie J. 2020. A universal CAR-NK cell targeting various epitopes of HIV-1 gp160. ACS Chem Biol 15:2299–2310. doi:10.1021/acschembio.0c00537 32667183 PMC8152219

[38] Maldini CR, Gayout K, Leibman RS, Dopkin DL, Mills JP, Shan X, Glover JA, Riley JL. 2020. HIV-resistant and HIV-specific CAR-modified CD4^+^ T cells mitigate HIV disease progression and confer CD4^+^ T cell help in vivo. Mol Ther 28:1585–1599. doi:10.1016/j.ymthe.2020.05.012 32454027 PMC7335752

[39] Zhen A, Carrillo MA, Mu W, Rezek V, Martin H, Hamid P, Chen ISY, Yang OO, Zack JA, Kitchen SG. 2021. Robust CAR-T memory formation and function via hematopoietic stem cell delivery. PLoS Pathog 17:e1009404. doi:10.1371/journal.ppat.1009404 33793675 PMC8016106

[40] Zhen A, Peterson CW, Carrillo MA, Reddy SS, Youn CS, Lam BB, Chang NY, Martin HA, Rick JW, Kim J, Neel NC, Rezek VK, Kamata M, Chen ISY, Zack JA, Kiem H-P, Kitchen SG. 2017. Long-term persistence and function of hematopoietic stem cell-derived chimeric antigen receptor T cells in a nonhuman primate model of HIV/AIDS. PLoS Pathog 13:e1006753. doi:10.1371/journal.ppat.1006753 29284044 PMC5746250

[41] Pampusch MS, Abdelaal HM, Cartwright EK, Molden JS, Davey BC, Sauve JD, Sevcik EN, Rendahl AK, Rakasz EG, Connick E, Berger EA, Skinner PJ. 2022. CAR/CXCR5-T cell immunotherapy is safe and potentially efficacious in promoting sustained remission of SIV infection. PLoS Pathog 18:e1009831. doi:10.1371/journal.ppat.1009831 35130312 PMC8853520

[42] Pampusch MS, Sevcik EN, Quinn ZE, Davey BC, Berg JM, Gorrell-Brown I, Abdelaal HM, Rakasz EG, Rendahl A, Skinner PJ. 2023. Assessment of anti-CD20 antibody pre-treatment for augmentation of CAR-T cell therapy in SIV-infected rhesus macaques. Front Immunol 14:1101446. doi:10.3389/fimmu.2023.1101446 36825014 PMC9941136

[43] Eichholz K, Fukazawa Y, Peterson CW, Haeseleer F, Medina M, Hoffmeister S, Duell DM, Varco-Merth BD, Dross S, Park H, Labriola CS, Axthelm MK, Murnane RD, Smedley JV, Jin L, Gong J, Rust BJ, Fuller DH, Kiem H-P, Picker LJ, Okoye AA, Corey L. 2024. Anti-PD-1 chimeric antigen receptor T cells efficiently target SIV-infected CD4^+^ T cells in germinal centers. J Clin Invest 134:e169309. doi:10.1172/JCI169309 38557496 PMC10977982

[44] Liu B, Zhang W, Xia B, Jing S, Du Y, Zou F, Li R, Lu L, Chen S, Li Y, Hu Q, Lin Y, Zhang Y, He Z, Zhang X, Chen X, Peng T, Tang X, Cai W, Pan T, Li L, Zhang H. 2021. Broadly neutralizing antibody-derived CAR T cells reduce viral reservoir in individuals infected with HIV-1. J Clin Invest 131:19. doi:10.1172/JCI150211 PMC8483761 34375315

[45] Chandra PK, Gerlach SL, Wu C, Khurana N, Swientoniewski LT, Abdel-Mageed AB, Li J, Braun SE, Mondal D. 2018. Mesenchymal stem cells are attracted to latent HIV-1-infected cells and enable virus reactivation via a non-canonical PI3K-NFκB signaling pathway. Sci Rep 8:14702. doi:10.1038/s41598-018-32657-y 30279437 PMC6168583

[46] University of Pennsylvania. 2019. A pilot study of T cells genetically modified by zinc finger nucleases SB-728mR and CD4 chimeric antigen receptor in HIV-infected subjects. https://clinicaltrials.gov/.

[47] Gibson L, Dooley S, Trzmielina S, Somasundaran M, Fisher D, Revello MG, Luzuriaga K. 2007. Cytomegalovirus (CMV) IE1- and pp65-specific CD8^+^ T cell responses broaden over time after primary CMV infection in infants. J Infect Dis 195:1789–1798. doi:10.1086/518042 17492595

[48] Janetzki S, Price L, Schroeder H, Britten CM, Welters MJP, Hoos A. 2015. Guidelines for the automated evaluation of Elispot assays. Nat Protoc 10:1098–1115. doi:10.1038/nprot.2015.068 26110715

[49] Grossman Z, Meier-Schellersheim M, Paul WE, Picker LJ. 2006. Pathogenesis of HIV infection: what the virus spares is as important as what it destroys. Nat Med 12:289–295. doi:10.1038/nm1380 16520776

[50] Monjure CJ, Tatum CD, Panganiban AT, Arainga M, Traina-Dorge V, Marx PA Jr, Didier ES. 2014. Optimization of PCR for quantification of simian immunodeficiency virus genomic RNA in plasma of rhesus macaques (Macaca mulatta) using armored RNA. J Med Primatol 43:31–43. doi:10.1111/jmp.12088 24266615 PMC3891828

[51] Rivière I, Brose K, Mulligan RC. 1995. Effects of retroviral vector design on expression of human adenosine deaminase in murine bone marrow transplant recipients engrafted with genetically modified cells. Proc Natl Acad Sci USA 92:6733–6737. doi:10.1073/pnas.92.15.6733 7624312 PMC41403

[52] Emtage PCR, Lo ASY, Gomes EM, Liu DL, Gonzalo-Daganzo RM, Junghans RP. 2008. Second-generation anti-carcinoembryonic antigen designer T cells resist activation-induced cell death, proliferate on tumor contact, secrete cytokines, and exhibit superior antitumor activity in vivo: a preclinical evaluation. Clin Cancer Res 14:8112–8122. doi:10.1158/1078-0432.CCR-07-4910 19088026 PMC2659496

[53] Wu C, Lu Y. 2007. Inclusion of high molecular weight dextran in calcium phosphate-mediated transfection significantly improves gene transfer efficiency. Cell Mol Biol (Noisy-le-grand) 53:67–74. doi:10.1170/T810 17531163 PMC2830788

[54] Kutner RH, Zhang XY, Reiser J. 2009. Production, concentration and titration of pseudotyped HIV-1-based lentiviral vectors. Nat Protoc 4:495–505. doi:10.1038/nprot.2009.22 19300443

